# Delay of Gratification Predicts Eating in the Absence of Hunger in Preschool-Aged Children

**DOI:** 10.3389/fpsyg.2021.650046

**Published:** 2021-03-19

**Authors:** Nicole R. Giuliani, Nichole R. Kelly

**Affiliations:** ^1^Department of Special Education and Clinical Sciences, Prevention Science Institute, University of Oregon, Eugene, OR, United States; ^2^Department of Counseling Psychology and Human Services, Prevention Science Institute, University of Oregon, Eugene, OR, United States

**Keywords:** self-regulation, eating in the absence of hunger, preschool, taste test, inhibitory control, delay of gratification

## Abstract

Poor ability to regulate one's own food intake based on hunger cues may encourage children to eat beyond satiety, leading to increased risk of diet-related diseases. Self-regulation has multiple forms, yet no one has directly measured the degree to which different domains of self-regulation predict overeating in young children. The present study investigated how three domains of self-regulation (i.e., appetitive self-regulation, inhibitory control, and attentional control) predicted eating in the absence of hunger (EAH) in a community sample of 47 preschool-aged children (*M* age = 4.93, SD = 0.86). Appetitive self-regulation, as measured using a delay of gratification task, was significantly and negatively associated with EAH 1 year later (*p* < 0.5). Measures of inhibitory and attentional control did not significantly predict EAH. These results suggest that food-related self-regulation may be a better predictor of overeating behaviors than general measures of self-regulation.

## Introduction

Developing healthy eating habits early in life is critical to establishing a healthy lifestyle and preventing the onset of diet-related diseases. Diet-related diseases once thought to be applicable only to adults (e.g., metabolic syndrome, type 2 diabetes, non-alcoholic fatty liver disease) are now being seen in children with increasing frequency (Daniels, 2006; Lucan, 2015). In the same way that individuals with a high body mass index (BMI) in childhood are more likely to continue to have a high BMI in adulthood (Guo et al., 2002), eating habits and food preferences established in childhood track into and through adulthood (Devine et al., 1998; Skinner et al., 2002; Nicklaus et al., 2004). As such, a better understanding of individual differences in eating behaviors related to high BMI and associated diseases is necessary to advance interventions aimed at improving health outcomes across the lifespan.

Many people eat not only in response to satiety, but also in response to external cues and emotions; these behaviors can lead to patterns of intake that go beyond energy needs (Dallman, 2010), increasing risk for diet-related diseases (Bleich et al., 2015). While often thought of in the context of adulthood, this phenomenon is also reliably seen in children and families (Blissett et al., 2010; Pieper and Laugero, 2013). The gold standard for measuring such eating behaviors in the laboratory is use of an “eating in the absence of hunger” paradigm (EAH; Birch et al., 2003), which measures the degree to which an individual continues consuming palatable foods beyond satiety. In children, EAH is associated with decreased satiety responsiveness (Carnell and Wardle, 2007) and greater adiposity (Cutting et al., 1999; Fisher and Birch, 2002; Hill et al., 2008; Zocca et al., 2011), both of which are related to an increased risk for elevated adult BMI and associated chronic diseases (Freedman et al., 2001; Juonala et al., 2011). Importantly, EAH has been successfully measured in children as young as 21 months (Asta et al., 2016), and has been used as a laboratory measurement of overeating in people of all ages (Fisher and Birch, 2002; Hill et al., 2008; Appelhans et al., 2011).

Extant data suggest that difficulties with self-regulation (Johnson and Birch, 1994; Disantis et al., 2011), may increase risk for children's tendency eat beyond satiety (McPhie et al., 2014). Self-regulation (SR) is defined as the ability to regulate one's own arousal, emotion, and behavior (Kopp, 1982; Bridgett et al., 2013). SR capacity relies on executive function (EF; Hofmann et al., 2012), a set of higher-level cognitive processes that support an individual's ability to regulate their behavior and emotion (Bridgett et al., 2013). Indeed, preschool-aged children with lower teacher-rated cognitive development scores have been shown to engage in more emotional-based EAH (Pieper and Laugero, 2013). While this study investigated and did not find an association between experimental tasks assessing EF and EAH, the authors acknowledged that their sample (*N* = 29) may have been too small to find such effects (Pieper and Laugero, 2013). Indeed, a broader literature on EF abilities has shown that it is meaningfully related to eating behaviors in preschool- (Allom and Mullan, 2014; Levitan et al., 2015; Reimann et al., 2020) and school-aged (Riggs et al., 2010a,b; Nederkoorn et al., 2015; Kelly et al., 2020) children (but see Hughes et al., 2015; Tan and Lumeng, 2018). A few studies have compared subdomains of EF (e.g., inhibitory control, updating), and suggest that they may be uniquely related to eating behavior (Allom and Mullan, 2014; Gettens and Gorin, 2017).

Like EF, SR is not a single construct. While work on SR and related constructs often conceptualizes them as unitary processes (e.g., Wiebe et al., 2011; Deater-Deckard, 2014), many models divide SR into different domains based on the degree of emotion involved (e.g., Metcalfe and Mischel, 1999; Willoughby et al., 2011; Bridgett et al., 2015). This multifaceted perspective on SR has been employed in the eating field, with most models separating out cool (i.e., solely behavioral) SR tasks from hot (i.e., emotional) SR tasks (e.g., Pieper and Laugero, 2013). One of the tasks used to assess hot SR is the classic delay of gratification paradigm (Willoughby et al., 2011), which requires individuals to control their desire to consume a single snack in order to gain a second snack. It may be that this process, which we refer to as “appetitive SR,” is conceptually more similar to self-regulating the desire to consume a tempting food in the absence of hunger as compared to more classic EF tasks or other forms of behavioral SR. However, no studies have directly compared appetitive and behavior SR with regard to eating in young children. Individual differences in SR abilities appear around age 3 (Carlson et al., 2004), and show dramatic growth through age 5 (Diamond, 2002). As such, the preschool period (defined here as aged 3 through 5) may be the ideal time to investigate the precise associations between SR and EAH in order to identify potential targets of intervention to alter developmental trajectories related to eating behaviors and the risk for associated diet-related diseases.

Therefore, in the present study we sought to investigate the associations between three separate forms of SR and EAH in a community sample of preschoolers. Appetitive SR was measured using a delay of gratification task, and two separate forms of behavioral SR were measured via attentional and inhibitory control tasks. We hypothesized that (1) all measured domains of SR would be inversely associated with EAH, such that greater SR ability would predict lower EAH, and (2) this association would be the strongest with regard to delay of gratification as compared to both forms of behavioral SR. Given past research suggesting that both delay of gratification and inhibitory control are associated with EAH, we ran additional exploratory analyses to examine whether interactions between appetitive and behavioral SR significantly predicted EAH.

## Methods

### Participants

The sample for the present study consisted of 47 preschoolers (*M* age at Session 2 = 4.93, SD = 0.86, range = 3.78–6.83 years) who participated in a follow-up session (hereby referred to as Session 2) following engagement in a larger study on SR in parents and children (hereby referred to as Session 1). Of the 89 families who participated in the larger study, 75 signed a consent form allowing the research team to recontact them for additional research opportunities. The subsample who returned ~1 year later for Session 2 did not differ from the full sample with regard to child age, sex, gross family income, maternal education, or maternal BMI (*p*-values > 0.28). Demographics for the present sample are detailed in Table 1.

**Table 1:** Demographic information.

**Demographics**	***M*** **(SD)**		**%**
**Child Demographics**	**Session 1**	**Session 2**	
Age (years)	4.00 (0.77)	4.93 (0.86)	
Female Race			49%
Caucasian			87.23%
Asian			2.13%
Hispanic			0%
Multiracial			8.51%
Native American/Indian			2.13%
Preschool attendance			61.7%
Household/parent demographics		
Mother highest level of education (years)	15.36 (2.46)		
Mother body mass index (kg/m^2^)	30.07 (8.01)		
Gross family income	$71,406.38 ($46,531.57)		

Families were recruited via physical and online flyers; criteria for participation were biological mothers over age 18 with children ages 3 through 5 who had not yet entered kindergarten at the time of Session 1. Exclusion criteria were if mothers had less than half-time custody of the child, had a history of significant neurological disorder, or were taking medication that affects cognitive function; if the child had a developmental delay, sensory impairment, or the mother believed the child could not participate in the study successfully; or if the family was involved with child welfare services or reported that their primary language was not English. There were no additional eligibility criteria to participate in Session 2. All study procedures were approved by the University's Committee for the Protection of Human Subjects.

### Protocol

In Session 1, mothers and children came into the laboratory for a roughly 3-h visit consisting of video-recorded parent-child interactions, mother-completed surveys, and child assessments of self-regulation, emotion identification, and school readiness. Measures relevant to the present analyses are described below. In Session 2, dyads returned to the same laboratory roughly 1 year later (*M* = 364.17 days, SD = 56.29) for a 2-h session scheduled around the time of day when mothers identified that the child usually ate lunch (all sessions began between 11 a.m. and 2 p.m., with 80.9% beginning at 11 a.m.). At the beginning of this session, mothers provided informed consent, after which both mother and child were weighed and measured for height in triplicate. Then, the child was presented with a 10,000 calorie test meal food array. Mothers were instructed to help their child eat lunch from the food array, but not eat anything themselves. These meals were video recorded. After lunch, mothers were asked to complete surveys while the child performed an EAH paradigm framed as a taste test in another room with the experimenter. Children were reunited with their mothers after 15 min. Families were then debriefed, thanked, and compensated $40 for their time.

### Measures

#### Family Demographics (Session 1)

At Session 1, mothers were asked to report the birth date, sex, and the race/ethnicity of their child. From that, age was calculated as the number of days between the child's birth and the session date, divided by 365.25. Mothers also reported the gross family income and her highest level of educational attainment by degree.

#### Anthropomorphic Measurements (Session 2)

Mother and child BMI were assessed using laboratory measurements of height (inches) and weight (pounds) at the beginning of Session 2. Individuals were asked to remove shoes and heavy clothing, and stand with their shoulders and heels against a wall. They were asked to take a breath in and out, and their height was measured using a stadiometer mounted on a flat wall at the exhale. This was done three times, and height (in inches) was calculated as the average of all measurements. Similarly, weight (in pounds) was measured three times using a digital scale and averaged. BMI was then calculated using the following equation: weight/height^2^ x 703. We converted BMI to z-score relative to same-age, same-sex peers (Mei et al., 2002) using Baylor College of Medicine's online BMI-percentile-for-age calculator (https://www.bcm.edu/cnrc-apps/bodycomp/bmiz2.html) for use in analyses.

#### Self-Regulation Tasks (Session 1)

##### Delay of Gratification Task

As detailed in Murray and Kochanska (2002), children were first asked to choose a preferred snack from an array of fruit snacks, M&Ms, and goldfish crackers. The experimenter placed the snack on a napkin in front of the children and asked them to wait until they rang a bell before retrieving it. The child was then told that they would receive a second snack if they were able to wait until the bell was rung. Four trials were conducted, where the child had to wait 30, 60, 120, and 180 s for the bell to ring. Halfway through each trial, the experimenter picked up the bell as if they were about to ring it. For each trial, the child was given a score representing waiting behavior: 0 (eats the snack before the bell is lifted), 1 (eats the snack after the bell is lifted), 2 (touches the bell or snack before the bell is lifted), 3 (touches the bell or snack after the bell is lifted), or 4 (waits for bell to ring before touching snack or bell). The final score was the average score over four trials, such that a child with an average score of 0 ate the snack before the bell was lifted for all trials, and a child with an average score of 4 waited until the bell was rung for all trials.

##### Flanker Task

The Flanker Task was administered via the NIH Toolbox Cognition Battery, which was adapted from the Attention Network Task (Rueda et al., 2004) and is normed for administration for children as young as 3 years old (Zelazo et al., 2014). Children were presented with a stimulus on the center of a tablet screen and were required to indicate the left-right orientation while inhibiting attention to the stimuli flanking it. On some trials the orientation of the flankers was congruent with the orientation of the central stimulus and on the other trials the flankers were incongruent. The test consisted of a block of 20 fish trials (designed to be more engaging and easier to see, and to make the task easier for children) and a block of 20 arrow trials, shown only if the participant scores >90% on the fish stimuli. The NIH Toolbox uses a two-vector method to score performance, which incorporated both accuracy and reaction time (RT) for participants who maintained a high level of accuracy (>80% correct), and accuracy only for those who did not meet this criterion. While age-referenced standardized scores are available for this task, we used raw scores in the present analyses in order to match the other SR tasks, for which age-referenced scores were not available.

##### Go/NoGo Task

Two GNG tasks were administered to children in the present study. First, children performed the Zoo Game (detailed in Grammer et al., 2014). Briefly, the task asked children to help a zookeeper put animals back in their cages by pressing a button as quickly as they can (Go [G] trials), unless they see Fred, a monkey who is helping the zookeeper (NoGo [NG] trials). The task began with three practice blocks in which children can practice (1) pressing the button on the laptop when they see an animal, (2) pressing the button within a certain time limit, and (3) practice inhibiting their response when they see the monkey. To increase the salience of the task, feedback was added at the end of each trial, such that children saw a smiling face if they correctly withheld their response on NG trials and a mad face if they either pressed the button on NG trials or did not press the button on G trials. Timing of this task was modified for the age range of the children in the present study by increasing the duration of the stimulus presentation and decreasing the number of trials. As such, each trial began with a 500–700 ms jittered fixation cross, 1,200 ms stimulus presentation, 500 ms black screen, and 1,000 ms feedback. Responses could be made while the stimulus was on the screen or at any point during the following 500 ms. A total of 90 trials were completed, 25% of which were NG. Percent correct was calculated across both types of trials.

We also asked children to complete the Fish GNG Task from the Early Years Toolbox (detailed in Howard and Okely, 2015). Briefly, the task asks children to respond to G trials (“catch fish,” 80% of trials) and withhold responding on NG trials (“avoid sharks,” 20% of trials). The task begins with go instructions followed by 5 practice go trials, no-go instructions followed by 5 practice no-go trials, combined GNG instructions followed by a mixed block of 10 practice trials (80% go trials), and a recap of instructions prior to the task commencing. Feedback in the form of auditory tones was provided on all practice trials. The task itself did not contain feedback, and was comprised of 75 test stimuli divided evenly into three test blocks (each separated by a short break and a reiteration of instructions). Stimuli were presented in pseudo-random order, such that a block never began with a no-go stimulus and no more than two successive trials were no-go stimuli, separated by a 1,000 ms interval between stimuli. Percent correct was calculated across both types of trials. Due to computer error, data from 15 participants were not recorded. Given the similarities in performance for the two GNG tasks (*r* = 0.439, *p* < 0.001), a composite score was created by z-scoring and averaging performance.

#### Test Meal (Session 2)

**Figure 1:**
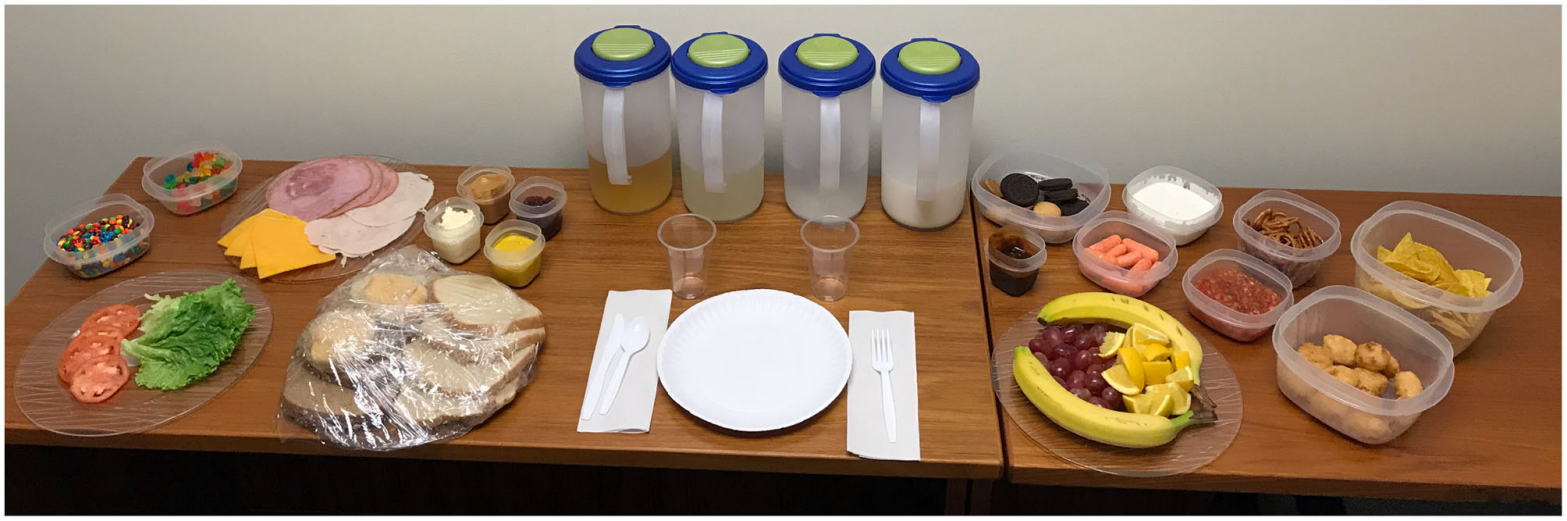
Test meal food array.

After anthropomorphic measurements were collected, mother-child dyads were escorted to a private room for lunch. They were instructed that the lunch was only for the child, but the mother was to help the child eat until they were no longer hungry. They were told that they had as much time as they needed, then granted access to a frequently used (Mirch et al., 2006; Tanofsky-Kraff et al., 2009; Shomaker et al., 2010a,b) *ad libitum* test meal varied in macronutrients (>10,000 kcal, Figure 1) and consisting of items most children like (e.g., bread, cheese, meat, chips, candy, cookies, fruit, chicken nuggets, water, milk, lemonade, apple juice). Mothers indicated before the session if there were any foods that should be omitted from the array due to allergies or vegetarian preferences (total *N* = 3; remove red food dye = 1, remove meat items = 2). All food items were weighed in grams (g) to the nearest single decimal before families entered the lunch room. When families completed lunch, experimenters ensured that they had not saved any food for later, and then weighed the remaining test meal food items when families were no longer able to see the lunch room. Energy content and macronutrient composition for each item were determined according to data from the USDA National Nutrient Database for Standard Reference, Release 24, and from the manufacturer labels on packaged food items. Total energy intake in kilocalories (kcal) was determined by subtracting the food weights after the participant's meal from premeal weights.

#### Eating in the Absence of Hunger (Session 2)

**Figure 2:**
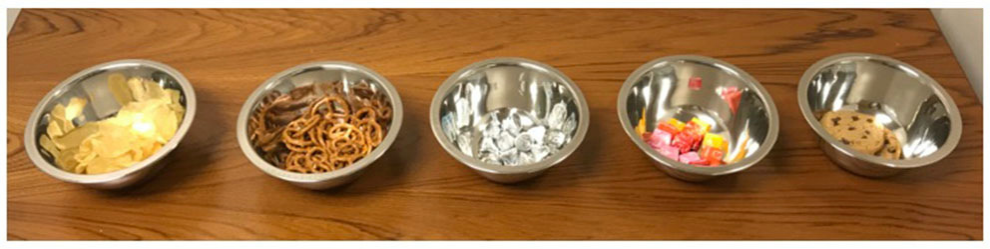
Taste test food array (from left to right: chips, pretzels, Hershey's kisses, Starburst, cookies).

Immediately after the completion of lunch, mothers were asked to complete a set of surveys in the waiting room, and children were escorted to a room containing the following foods displayed in separate bowls (Figure 2): potato chips (28g; Kettle brand Sea Salt), pretzel twists (28g; Rold Gold brand Tiny Twists), chocolate drops (90g, Hershey brand kisses, individual-wrapped), fruit chew candies (150g; Starbursts brand, individually-wrapped), and chocolate chip cookies (70g; Grandma's brand). Mothers had indicated which foods their child should not eat due to allergies beforehand; children performed their taste test using only the foods that were permitted by their mothers (total N = 1; removed red/pink Starburst containing red food dye). Consistent with the original paradigm used in the proposed age range (Cutting et al., 1999; Fisher and Birch, 1999), children were instructed to taste each of the foods and provide a rating from 1 to 5 using a smiling-face scale where 1 = “very tasty” and 5 = “not very tasty” validated for use in the assessment of taste in pediatric populations (Mistry et al., 2018). Children were encouraged to complete the taste test within 5 minutes, and were then told that they had to remain in the room while their mother completed her surveys. They were told that they could eat as much of the remaining food as they wanted, as well as play with a bin of toys in the room opened by the experimenter. The experimenter remained in the room with the child for the full duration of the taste test and subsequent play period, and was instructed to minimize interactions with the child. After 15 minutes, the child was escorted to their mother. EAH was measured by calculating the difference in weight (g) of each snack before and after the eating period and summing across all snacks. Energy intake was calculated using the same methods as for the test meal.

### Analyses

Study variables were assessed for skew and kurtosis; variables with a skewness or kurtosis over ±1 were transformed to improve distributions and re-assessed. Total calories consumed during lunch was identified as non-normally distributed. The distribution of this variable was greatly improved by transformation using the transform Tukey function in the R package *rcompanion* (Mangiafico, 2019), which follows the Tukey's Ladder of Powers principle to improve the distribution of skewed variables. This transformed variable was used for all subsequent analyses. A missing data analysis revealed that 7 participants were missing data from the Flanker task, and 2 were missing data from the GNG tasks. The majority of the data points lost were due to a computer error, which is considered to be missing completely at random. Therefore, we imputed all the missing data using multiple imputation implemented using the *mice* package in R (van Buuren and Groothuis-Oudshoorn, 2011).

All analyses were run using R (R Core Team, 2019). Zero-order associations between scores on the three SR tasks were first run using Pearson's correlations, adjusted for multiple tests using the Benjamini-Hochberg correction (Benjamini and Hochberg, 1995); adjusted *p*-values are presented. Associations between SR and EAH were tested using three separate linear regression models, one for each form of SR. To explore the interactions between the different forms of SR on EAH, we entered all three forms of SR in the same model and tested two- and three-way interactions between SR tasks. Interactions were interrogated and plotted using the R package *interactions* (Long, 2019). Covariates in all models included child BMI z-score (Session 2) and total calories consumed (kcal) during the test meal (Session 2). Confirmatory analyses were also analyzed using % estimated energy requirements (calculated according to the Institute of Medicine guidelines; Institute of Medicine of the National Academies, 2005); the pattern of results did not change.

## Results

### Confirmatory Results

As shown in Table 2, zero-order correlations between SR tasks revealed that delay of gratification (as measured by Snack Delay score) was not significantly associated with either attentional control (as measured by the Flanker Task), *r*_(45)_ = 0.22, *p* = 0.16, or inhibitory control (as measured by the Go/NoGo composite), *r*_(45)_ = 0.13, *p* = 0.48. Attentional and inhibitory control were significantly positively associated, *r*_(45)_ = 0.43, *p* = 0.01.

**Table 2:** Descriptive data of self-regulation, test meal, and EAH variables, and correlations with confidence intervals.

**Variable**	***M***	***SD***	**Range**	**1**	**2**	**3**	**4**
1. Snack Delay Task	1.80	1.71	0–4				
2. Flanker Task (raw score)	20.43	12.07	4–40	0.22			
				[−0.09, 0.48]			
3. Go/NoGo Task composite	0.04	0.76	−2.12–1.62	0.11	0.44^**^		
				[−0.19, 0.40]	[0.13, 0.67]		
4. Test meal (total kcal consumed)	492.57	300.14	128.54–1351.30	−0.03	0.37^*^	0.11	
				[−0.32, 0.26]	[0.09, 0.60]	[−0.19, 0.39]	
5. EAH (total kcal consumed)	120.90	72.11	27.64–319.99	−0.27	0.15	0.18	0.37^**^
				[−0.52, 0.01]	[−0.16, 0.44]	[−0.11, 0.45]	[0.10, 0.60]

M and SD are used to represent mean and standard deviation, respectively. Correlations were run on the pooled estimates from multiply imputed data sets. Values in square brackets indicate the 95% confidence interval for each correlation. The confidence interval is a plausible range of population correlations that could have caused the sample correlation (Cumming, 2014). EAH, eating in the absence of hunger.

* *Indicates p < 0.05*.

** *Indicates p < 0.01*.

**Figure 3:**
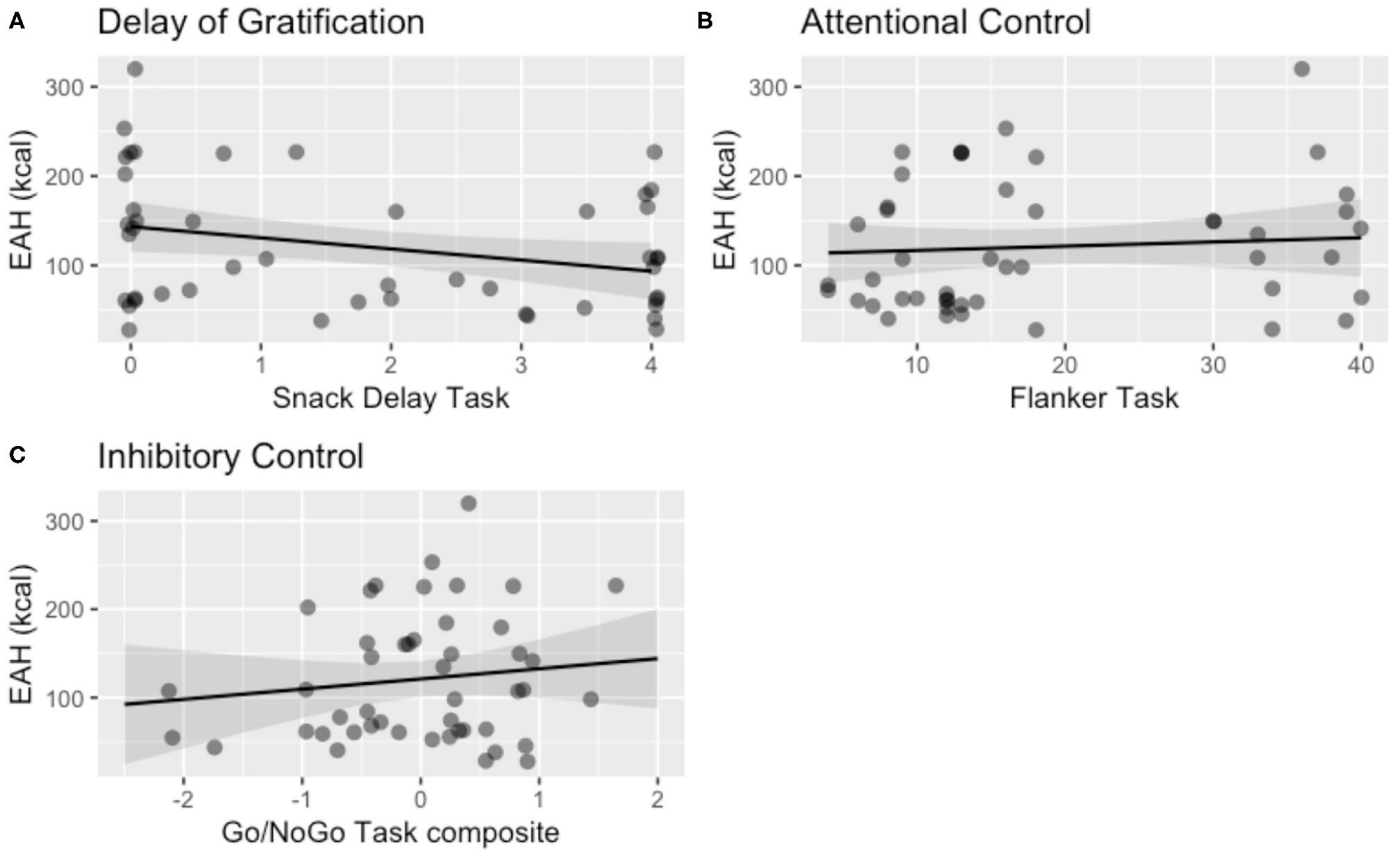
Visualization of the main effect of child self-regulation on eating in the absence of hunger (EAH) controlling for child age, child sex, child BMI, and the number of calories (kcal) consumed during the lunch test meal, in the domains of **(A)** delay of gratification (*b* = −12.46, *p* = 0.04), **(B)** attentional control (*p* = 0.67), and **(C)** inhibitory control (*p* = 0.34). One of the multiply imputed data sets was chosen at random for plotting purposes.

Delay of gratification at Session 1 was negatively associated with total calories consumed (kcal) during the EAH paradigm 1 year later at Session 2, *b* = −12.46, 95% CI [−23.95, −0.97], SE = 5.86, *t*_(41.13)_ = −2.13, *p* = 0.040 (Table 3A). Attentional control at Session 1 was not associated with EAH at Session 2, *b* = 0.38, 95% CI [−1.59, 2.34], SE = 1.03, *t*_(33.38)_ = 0.37, *p* = 0.71 (Table 3B), nor was inhibitory control, *b* = 13.50, 95% CI [−13.33, 40.34], SE = 13.69, *t*_(40.94)_ = 0.99, *p* = 0.33 (Table 3C). Visualization of these results for total calories consumed are shown in Figure 3.

**Table 3:** Results of the multiple regression analyses by self-regulation domain.

**Predictor**	***t***	***p***	***b***	***R^**2**^***
(A) Appetitive self-regulation				0.185
Intercept	−2.195	0.034	−1493.736^*^	
Snack Delay	−2.126	0.040	−12.462^*^	
Child BMIz	−0.670	0.507	−8.224	
Kcal consumed at lunch^‡^	2.405	0.021	1410.550^*^	
(B) Attentional control				0.105
Intercept	−1.808	0.078	−1350.670	
Flanker Task	0.375	0.710	0.376	
Child BMIz	−0.442	0.661	−5.675	
Kcal consumed at lunch^‡^	1.945	0.059	1261.262	
(C) Inhibitory control				0.120
Intercept	−1.852	0.071	−1324.944	
Go/NoGo Task composite	0.986	0.330	13.502	
Child BMIz	−0.193	0.848	−2.499	
Kcal consumed at lunch^‡^	2.019	0.050	1244.224	

*The dependent variable for all regressions was EAH, defined as total calories (kcal) consumed during the taste test. BMIz = z-scored body mass index. All parameters were calculated using pooled estimates from multiply imputed data sets*.

‡ *Variable transformed*.

* *p < 0.05*.

**Figure 4:**
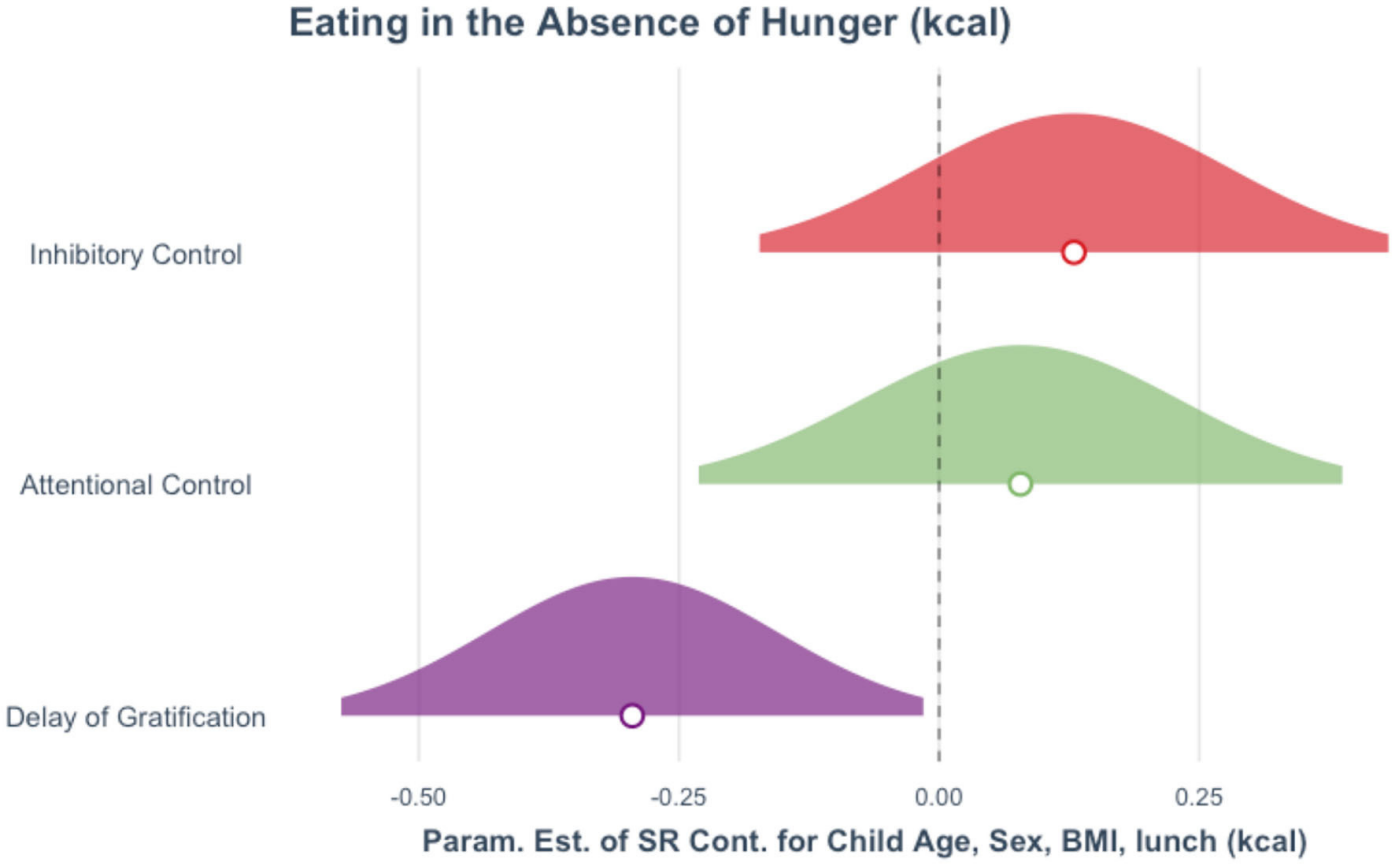
Visualization of the effects of self-regulation on eating in the absence of hunger (EAH) by self-regulation (SR) domain, controlling for child age, child sex, child BMI, and the number of calories (kcal) consumed during the lunch test meal. Each curve represents the 95% confidence interval, circles represent the standardized parameter estimates for inhibitory control (ß = 0.130), attentional control (ß = 0.078), and delay of gratification (ß = −0.295). One of the multiply imputed data sets was chosen at random for plotting purposes.

A direct comparison of the confidence intervals for the effects of SR on EAH by domain revealed that, while the confidence intervals overlapped (Figure 4), the 95% confidence interval for delay of gratification did not include the estimated associations of attentional and inhibitory control with EAH. We compared standardized regression coefficients using Eid et al.'s (2011) formulas implemented in the Psychometrica online calculator (Lenhard and Lenhard, 2014), which revealed that the effect of delay of gratification on EAH was indeed significantly higher than the effect of inhibitory control, *z* = −2.15, *p* = 0.016, but not attentional control, *z* = −1.35, *p* = 0.089. An exploratory direct comparison of the tasks assessing all three SR domains in the same model revealed that no one SR domain was significantly associated with EAH when controlling for the other two SR domains as well as child BMI z-score and total calories consumed during lunch (*p*-values > 0.11). Full models, data, and R scripts are available online – https://osf.io/wbntq/.

### Exploratory Results

**Figure 5:**
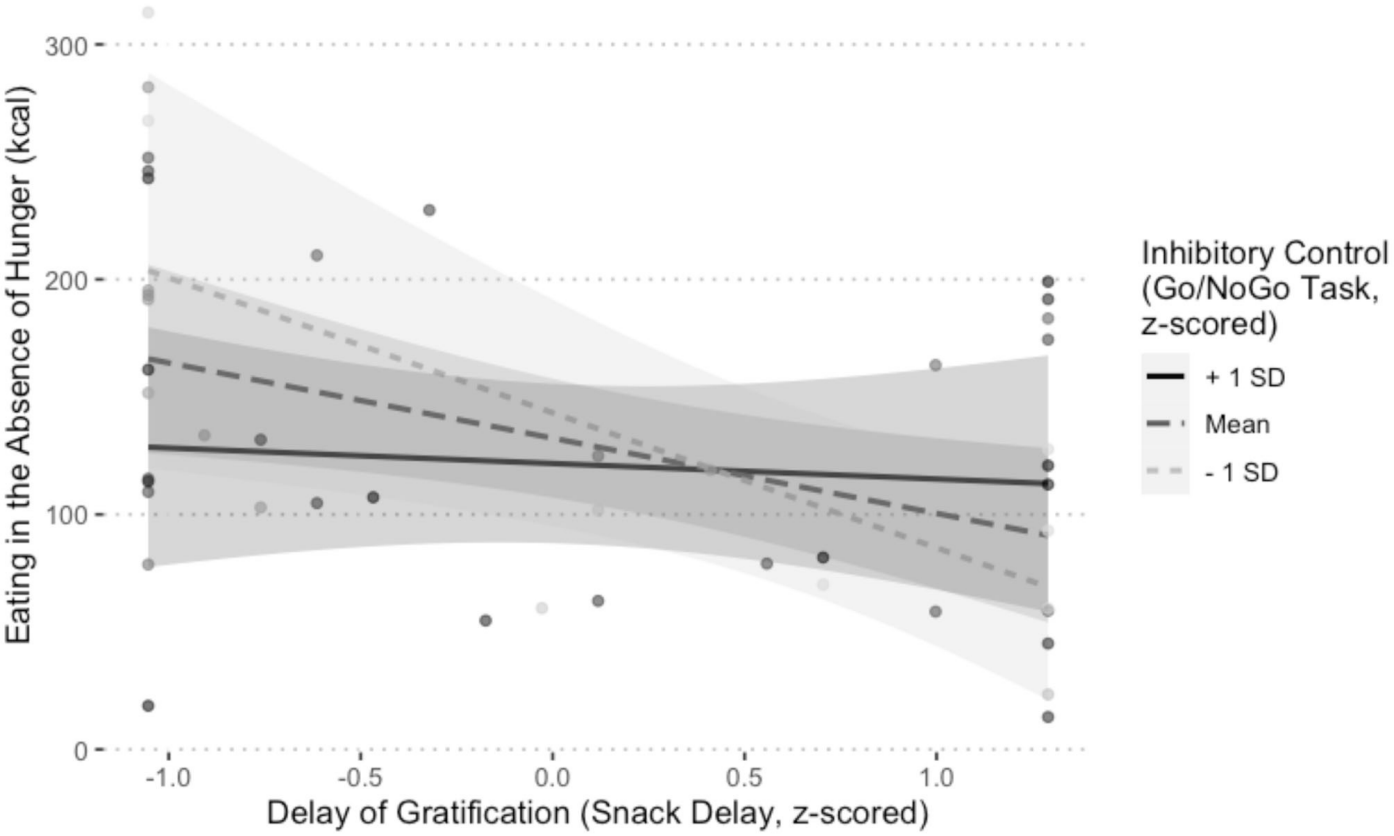
Visualization of the interaction between inhibitory control (Go/NoGo Task) and delay of gratification (Snack Delay Task) on eating in the absence of hunger (EAH), controlling for child age, child sex, child BMI, and the number of calories (kcal) consumed during the lunch test meal, *b* = 42.22, *p* = 0.04. One of the multiply imputed data sets was chosen at random for plotting purposes.

As shown in Table 4, there was a significant interaction between delay of gratification and inhibitory control at Session 1 on EAH at Session 2, *b* = 42.22, 95% CI [3.85, 80.58], SE = 19.57, *t*_(27.18)_ = 2.16, *p* = 0.04. Simple slopes analyses performed on one of the multiply imputed data sets revealed that the slope of the association between delay of gratification and calories consumed was significant for individuals who performed worse than −1 SD below or at the mean on inhibitory control (−1 SD: *b* = −57.46, SE = 21.62, *t* = −2.66, *p* = 0.01; mean: *b* = −32.03, SE = 12.44, *t* = −2.57, *p* = 0.01). In other words, children who were at or below the mean on both the Snack Delay and Go/NoGo Tasks consumed the most calories (Figure 5). All other interactions were non-significant (*p*-values > 0.11).

**Table 4:** Results of the multiple regression analyses examining interactions between self-regulation domains.

**Predictor**	***t***	***p***	***b***	***R^**2**^***
				0.325
Intercept	−2.198	0.037	−1796.711	
Snack Delay (z-scored)	−2.241	0.033	−32.180^*^	
Flanker (z-scored)	1.561	0.130	24.877	
GNG (z-scored)	−1.366	0.183	−27.139	
Child BMIz	−1.243	0.225	−18.717	
Kcal consumed at lunch^‡^	2.366	0.025	1663.941^*^	
Snack^*^Flanker	−1.680	0.105	−25.787	
Snack^*^GNG	2.157	0.040	42.217^*^	
Flanker^*^GNG	−0.811	0.425	−14.911	
Snack^*^Flanker^*^GNG	0.414	0.682	7.483	

*The dependent variable for all regressions was the total calories (kcal) consumed during the taste test. Snack, Snack Delay Task score; GNG, Go/NoGo Task composite variable; BMIz, z-scored body mass index. All parameters were calculated using pooled estimates from multiply imputed data sets*.

‡ *Variable transformed*.

* *p < 0.05*.

## Discussion

In this study, we first hypothesized that SR would inversely predict EAH ~1 year later in a community population of preschool-aged children. In partial support of this hypothesis, we found that there was a significant negative association between Snack Delay Task score in Session 1 and total calories consumed during the taste test at Session 2. Children who were able to wait until the end of all delay periods on the Snack Delay Task consumed, on average, approximately 50 calories fewer than children who were unable to wait during any of the delay periods. There was no significant association between SR and EAH in the domains of attentional or inhibitory control (*p*-values > 0.33).

Our second hypothesis was that the association between SR and EAH would be strongest in the domain of appetitive SR, such that delay of gratification be a better predictor of EAH as compared to inhibitory and attentional control. In support of this hypothesis, we found that the only significant effect of SR on later EAH was in the domain of appetitive SR, and a direct comparison of the standardized regression coefficients revealed that the effect of delay of gratification on EAH was indeed significantly higher than the effect of inhibitory control (the comparison with attentional control was at the trend level). However, the effect of delay of gratification on EAH was not significant when controlling for attentional and inhibitory control. This is most likely due to a combination of reduce degrees of freedom with an already moderate sample size, as well as the shared variance between the three SR tasks (see Table 2). Therefore, while delay of gratification performance on the Snack Delay Task is a significant predictor of later EAH, we are unable to use these data to definitively conclude that it is a better predictor compared to other measures of SR.

Taken in context with the literature on SR and eating behavior, future research should examine how the link between SR and EAH changes over time. SR-related skills are some of the last neurocognitive skills to fully develop and each domain appears to grow at a different pace (Brocki and Bohlin, 2004; Casey et al., 2005; Huizinga et al., 2006). However, most of these studies do not include children as young as those in the current study. A recent review of the SR literature separating food and non-food SR in early childhood concluded that, while there are suggestions of common underpinnings of both forms of SR, each domain develops somewhat independently with increasing integration across childhood (Russell and Russell, 2020). The present findings that delay of gratification was not significantly associated with either attentional or inhibitory control in children aged 3–6 fits within this framework. As such, longitudinal studies of associations with pediatric EAH are warranted. Interventions aimed at improving eating habits should be developed in age-appropriate ways, including the relative SR domain development of the target population.

This study had some limitations. First, only 47 of the 75 families we contacted participated in Session 2. While these families did not meaningfully differ from the full set of families with regard to demographics, there may be other differences that we did not capture. Second, because these were secondary analyses, we did not run *a priori* power analyses to determine the necessary sample size to achieve appropriate statistical power to test our hypotheses. A *post hoc* sensitivity analysis in G^*^Power (Faul et al., 2009) revealed that the present sample size of 47 was powered (α = 0.05, power = 0.8) to detect small-to-medium effect sizes (*f*^2^ = 0.18). The present findings found a small effect of delay of gratification on EAH (*f*^2^ = 0.12), and thus should be interpreted with caution. Third, the composition of the participants in this study was relatively homogeneous with regard to race and ethnicity; as such these results may not be generalizable to other racial/ethnic groups. These families were not recruited based on obesity risk, and were limited with regard to child BMI. We also limited our sample to biological mothers to reduce caregiving variance, which additionally reduces the generalizability of these findings. Fourth, families were told that their meals were being video recorded. While the cameras were unobtrusively placed in the room, this may have affected how much the child ate or how the mother fed the child. Fifth, while we asked families to join us during their typical lunch time, we do not have information as to what the children ate prior to the laboratory session. Lastly, as they were done in controlled laboratory settings, the test meal and taste test protocols may not fully approximate eating behavior in the real world.

The purpose of this study was to quantify the degree to which SR in preschool-aged children predicted EAH across three different domains of SR. While previous studies have documented a link between EF and eating behaviors associated with increased weight and risk of diet-related diseases (e.g., Allom and Mullan, 2014; Levitan et al., 2015; Reimann et al., 2020), this is the first time that different domains of SR have been directly compared in the same sample of preschool-aged children. We found that appetitive SR, as measured by performance on a delay of gratification task, was significantly negatively associated with EAH about 1 year later. Performance on inhibitory and attentional control tasks was not. There was also a significant interaction between appetitive SR and inhibitory control, such that children who evinced poor performance on the tasks assessing both forms of SR ate a greater number of calories during the EAH session than other children. These results support previous findings that self-regulation is meaningfully associated with eating behavior, but suggest that these effects may be strongest in the domain of appetitive self-regulation.

## Data Availability Statement

The raw data supporting the conclusions of this article will be made available by the authors, without undue reservation.

## Ethics Statement

The studies involving human participants were reviewed and approved by University of Oregon Institutional Review Board. Written informed consent to participate in this study was provided by the participants' legal guardian/next of kin.

## Author Contributions

NG and NK designed the study, edited drafts, and approved the final version. NG collected and analyzed the data and wrote the manuscript. All authors contributed to the article and approved the submitted version.

## Conflict of Interest

The authors declare that the research was conducted in the absence of any commercial or financial relationships that could be construed as a potential conflict of interest.
